# Role of Macrophage Migration Inhibitory Factor (MIF) in Melanoma

**DOI:** 10.3390/cancers11040529

**Published:** 2019-04-12

**Authors:** Laura Soumoy, Nadège Kindt, Ghanem Ghanem, Sven Saussez, Fabrice Journe

**Affiliations:** 1 Department of Human Anatomy and Experimental Oncology, Université de Mons (UMons), Research Institute for Health Sciences and Technology, 7000 Mons, Belgium; laura.soumoy@umons.ac.be (L.S.); nadege.kindt@umons.ac.be (N.K.); sven.saussez@umons.ac.be (S.S.); 2 Laboratory of Oncology and Experimental Surgery, Institut Jules Bordet, Université Libre de Bruxelles (ULB), 1000 Brussels, Belgium; gghanem@ulb.ac.be; 3 Department of Oto-Rhino-Laryngology, Université Libre de Bruxelles (ULB), CHU Saint-Pierre, 1000 Brussels, Belgium

**Keywords:** melanoma, MIF, immunity, metastasis, combined therapies

## Abstract

Macrophage migration inhibitory factor (MIF) is an inflammatory cytokine involved in the carcinogenesis of many cancer types. Here, we review the published experimental and clinical data for MIF and its involvement in melanoma. All reported data show that MIF is overexpressed in melanoma cells, especially in case of metastatic disease. Clinical studies also indicate that high MIF expression is positively associated with aggressiveness of the disease. Some data also highlight the implication of MIF in angiogenesis, immunity and metastasis in melanoma cell lines, as well as the availability of different therapeutic options targeting MIF for the treatment of metastatic melanoma. Indeed, the main problem in metastatic melanoma is the lack of long-term effective treatment. This is linked to the capacity of melanoma cells to mutate very quickly and/or activate alternative signaling pathways. Thus, MIF targeting therapies could provide a new effective way of treating melanoma. Moreover, cell sensitivity to MIF depletion does not correlate with the BRAF mutational status. Regarding the fact that many melanoma patients carry a BRAF mutation, and that they develop resistance to BRAF inhibitors, this observation is very interesting as MIF inhibitors could be used to treat many patients in relapse after treatment with an inhibitor of the mutant BRAF protein.

## 1. Introduction

### 1.1. Melanoma

Melanoma is the most common and deadliest form of skin cancer. This type of tumor is affecting an increasing number of young adults. Indeed, melanoma represents the first form of cancer among people aged 25 to 29 [1]. This disease is particularly difficult to treat, especially when diagnosed at a late stage. Most melanoma cells are radio and chemo-resistant, mainly due to their melanin production. The current treatments for metastatic melanoma rely on targeted therapies and, more recently, immunotherapies. The most used targeted therapies act on the MAPK pathway, which is mutated in NRAS and BRAF in about 25 and 60% of melanoma patients, respectively [2]. Nevertheless, the major challenge with these kinds of inhibitors is that melanoma cells possess an hypermutable genome and many alternative signaling pathways, leading to resistances to such therapies [3], further supporting the use of a combination of treatments [1].

### 1.2. MIF and Cancer

Macrophage migration inhibitory factor (MIF) is a pro-inflammatory cytokine which inhibits the random movement of macrophages. This factor was first described as released by T lymphocytes [4], but several studies have since shown that MIF is synthesized by many other cell types, such as epithelial cells, endothelial cells and macrophages [5].

It has long been known that MIF is implicated in inflammatory diseases including atherosclerosis, systemic lupus erythematosus, psoriasis and diabetes [6,7]. In recent years, several studies have demonstrated that an overexpression of MIF occurs in many tumors, including lung, colorectal, breast, prostate, and head and neck cancers. Globally, its overexpression seems to play a key role in tumor progression by regulating both cell proliferation and invasiveness [8,9,10,11,12]. In this context, MIF is therefore discussed as a promising target for therapies.

MIF plays its oncogenic role in autocrine and paracrine manners. It has been noted that the effects of MIF in cancer mainly occur through its binding to the CD74 receptor [13], even if other receptors, such as the chemokine receptor CXCR4, or the CD44 receptor, have also been reported to be involved in MIF signaling [14].

### 1.3. Genomic Alteration of MIF in Melanoma

Genomic alterations of MIF, CD74 and CD44 in skin cutaneous melanoma have been investigated based on The Cancer Genome Atlas data sets. For the purpose of this study, we analyzed the rate of mutations and copy number variations in 479 melanomas using the cBioPortal for Cancer Genomics (http://www.cbioportal.org/). We found molecular alterations in only 1% of cases for MIF, 3% for CD74, and 2.4% for CD44, mainly including gene amplifications and missense mutations.

### 1.4. MIF mRNA and Protein Expression in Melanoma

In 1999, Shimizu et al. [15] reported, for the first time, that human melanocytes and melanoma cells express MIF mRNA and produce MIF protein. Their Northern and Western blot analyses showed that the expression of MIF mRNA and the production of MIF protein were much higher in human melanoma cell lines than in normal cultured melanocytes [15].

Moreover, cDNA array analysis and Northern blotting performed by Rumpler et al. [16] showed that MIF is overexpressed in the melanoma cell lines harboring an aggressive phenotype (high proliferative and migratory potential). They also showed that the expression of MIF in cutaneous melanoma was three times higher in metastases than in primary lesions [16].

A study comparing MIF expression in different cutaneous melanocytic tumors reported that MIF protein is highly produced in both benign and malignant lesions, but that MIF mRNA expression is significantly higher in malignant ones, suggesting either a difference in translational regulation or protein stability. They also showed that the subcellular localization differs in benign and malignant lesions. Indeed, MIF protein presented a homogeneous cytoplasmic expression in benign lesions, while the cytoplasmic expression was more heterogeneous in malignant ones. They also observed a higher number of cell nuclei expressing MIF protein in cancers than in benign lesions, assuming a role for MIF nuclear location in tumorigenesis [17]. A clinical study analyzed the MIF level in the serum of melanoma patients. The results revealed that the MIF amount in serum is higher in melanoma patients than in healthy controls [18].

Altogether, many in vitro studies have proven that MIF expression is higher in melanoma cells, especially in case of aggressive disease. This MIF overexpression has also been shown in vivo and in patient samples. Translational regulation mechanism(s) and impact of MIF localization on the melanoma progression still need to be further studied. However, an explanation for its implication in cancer could be linked to the role of Hsp90. Indeed, in many cancer types, MIF has been shown to be highly stabilized by Hsp90 [19].

### 1.5. MIF and Pigmentation

A comparison performed by studying the transcriptional profiles of melanocytes from dark and light skinned individuals showed that MIF is a regulator of melanogenesis. Indeed, immunohistochemistry indicated that MIF expression was high in the cytoplasm of melanocytes with many dendrites and, consequently, well-differentiated cells.

They also studied the impact of UV on MIF expression, and showed that MIF was also liberated from melanocytes when low UV doses were used to stimulate them. As such, it emerged from this study that MIF expression varies physiologically according to the patient’s skin pigmentation and the environmental conditions [20].

Finally, a previous study reported that MIF exhibits a nonphysiologic dopachrome tautomerase activity which has been extensively exploited to screen potential inhibitors, since compounds antagonizing this enzymatic activity also disrupt MIF interactions with its cognate receptor CD74 [21]. We may hypothesize that such MIF activity could also be involved in melanogenesis as dopachrome tautomerase (DCT) is a melanogenic protein involved in the pigmentation of human melanocytes [22].

## 2. MIF Impact on Melanomagenesis

### 2.1. MIF and Melanoma Patient Outcome

A study performed at the University of Texas on metastasis of melanoma patients with stage III disease indicated that the presence of MIF protein expression in tumors was associated with poor survival parameters in both overall survival and relapse-free survival. Intriguingly, in melanomas, the expression of CD74, the main receptor of MIF, correlated with a favorable patient outcome. Furthermore, their study indicated that the signature CD74^+^/MIF^−^ could be a more powerful prognostic marker in the case of stage III melanoma [22].

Nevertheless, MIF expression can only be used as a prognosis marker in the case of metastatic melanoma. Indeed, analysis performed by Oliveira et al. [23] showed that MIF expression levels were not associated with patient outcome in primary melanoma, but higher MIF levels were correlated with faster disease recurrence in metastatic disease [24], correlating with the results of the Ekmekcioglu study [24].

### 2.2. Signaling Pathways

**Figure 1:**
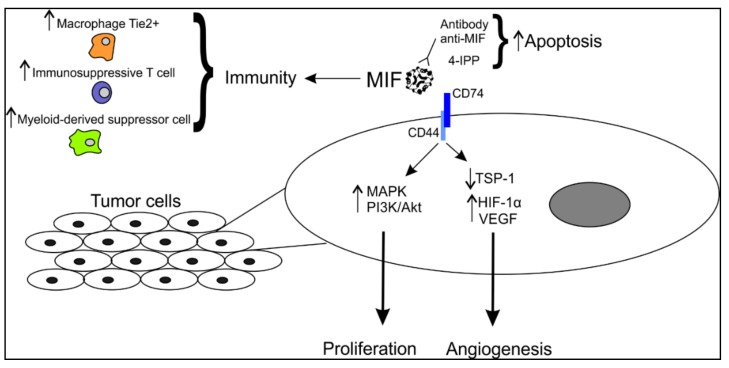
Schematic representation of the involvement of MIF in melanoma cells. MIF is secreted in the microenvironment and can interact with tumor cells via its membrane receptor CD74. In this way, it affects several pathways in proliferation and angiogenesis. MIF can also modulate the anti-tumor immune response by upregulating the expression of specific immune cells playing an immunosuppressive role. MIF, migration inhibitory factor; MAPK, mitogen-activated protein kinase; PI3K, phosphoinositide 3-kinase; TSP-1, thrombospondin 1; HIF-1α, hypoxia-induced factor 1α; VEGF, vascular endothelial growth factor.

Depending on the cellular context, MIF can bind to different receptors, such as CD74, the first identified and the most often involved in MIF signaling, CXCR2 or CXCR4 [23] (Figure 1).

Most of the time, MIF acts by binding to CD74, which forms a complex with the co-receptor CD44 on the cell surface. This binding triggers the sustained activation of the MAPK and the PI3K/AKT pathways, which are frequently upregulated in melanomas through NRAS or BRAF mutation, or PTEN loss, respectively. In vitro analysis conducted in human melanoma cell lines showed that MIF acts upstream of the PI3K/AKT pathway, as MIF knock-down decreased AKT phosphorylation. A correlation between the degree of sensitivity of cell lines to MIF depletion and the inhibition of AKT activity has also been reported. Notably, the effect of MIF on AKT leads to modifications in the proteins regulated by its pathway, including a decrease of cyclin D1 and CDK4 expression, as well as an increase of the p27 level. Altogether, these effects act by retarding the cell cycle progression leading to more cells in G1/S transition and less cells in S phase [25].

There is another important issue concerning the role of the CD74 in melanoma. Indeed, CD74 expression is associated with melanoma progression but, as previously seen (Section 2.1), high levels of CD74 in melanoma metastasis are associated with a better overall and recurrence-free survival, in the absence of MIF in the tumor microenvironment. This can be explained by the fact that CD74 acts in at least two ways. This receptor links to MIF, but it also plays a role in MHC processing and potentiates antigen expression in tumors. In this way, CD74 has a potential role in anti-tumor immune responses [24].

In addition, Nguyen et al. [26] showed that MIF is implicated in the regulation of cellular redox stress and can inhibit oxydative stress-induced apoptosis. As melanoma is a cancer characterized by an altered redox status, these observations were crucial to understand the role of MIF in such disease [26].

### 2.3. Angiogenesis

It is a well-known fact that MIF is implicated in angiogenesis in many cancers. Indeed, it has been reported that MIF production by melanoma cells leads to an induction of tube formation and the migration of endothelial cells, leading to the formation of new blood vessels in the tumor microenvironment [27].

Many in vivo studies have shown a link between MIF production by melanoma cells and angiogenesis in melanoma models. For example, the treatment of melanoma bearing mice with anti-MIF antibodies led to a significantly reduced angiogenesis [15]. Another study using an interfering MIF RNA introduced into melanoma cell lines showed a significantly delayed tumor development linked to a marked absence of intra-tumoral vascularization. These tumors also had a similar size while measured horizontally but not vertically [28]. This last observation is crucial, knowing that it is the vertical growth phase that leads to the development of metastasis. In their study, Culp et al. [28] used the B16-F10 melanoma cell line model in which MIF expression was inhibited to demonstrate that MIF produced by melanoma cells acts on angiogenesis by downregulating the expression of the anti-angiogenic factor thrombospondin 1 (TSP-1), which inhibits endothelial cell migration [29]. In addition, MIF can also be produced by the surrounding stroma and act on other aspects of angiogenesis. Indeed, regarding the results of Girard’s studies [30], MIF ability to regulate tumor vascularization could also be linked to its ability to regulate the expression of pro-angiogenic chemokines and to modulate the recruitment and activity of pro-inflammatory cells.

Both MIF produced by melanoma cells as well as MIF produced by the stroma are also implied in tumor angiogenesis by acting on other signaling pathways, including the hypoxia-induced factor HIF-1α and the vascular endothelial growth factor VEGF. Indeed, MIF enhances Jab1/CSN5-dependent stabilization of HIF-1α which leads to the activation of hypoxia-responsive genes [31]. In vitro studies on melanoma cell lines also showed that MIF knock-down resulted in a reduced expression of VEGF, and this was associated with a decreased response to hypoxia. Altogether, these data demonstrate that both tumor cell-derived MIF and circulating MIF could play major local and systemic roles in angiogenesis promotion, respectively. Actually, in vivo experiments on wild-type and MIF^−/−^ mice, in which MIF deficient tumor cells were implanted, showed a decreased angiogenesis in the MIF^−^/^−^ mice but not in the wild-type individuals [30].

All these studies have allowed to establish the crucial influence of MIF on angiogenesis. Indeed, MIF has many ways to modulate angiogenesis by acting on many factors implied in this phenomenon, such as the anti-angiogenic factor TSP-1, the growth factor VEGF, pro-angiogenic chemokines, and the transcription factor HIF-1α, which modulates the activity of hypoxia responsive genes, as well as pro-inflammatory cells (Figure 1).

Previous studies have shown the role of MIF on angiogenesis in primary tumors, but an effect on metastasis formation has also been reported. Indeed, it has been shown that MIF promotes macrophage angiogenic potential by acting on tumor-associated macrophage (TAM) polarization in lung metastases [32].

Furthermore, the angiogenic pathway has been shown to involve the concerted activities of not only vascular elements but also myeloid cells. This shows that MIF acts on cells implied in immunity, and influences many pathways, such as the immune response, and impacts the mechanisms of proliferation and angiogenesis [32,33].

Regarding the modulation of angiogenesis by MIF, targeting this factor could be effective in slowing down tumor development by reducing the blood supply to the primary lesion, and then reducing metastasis development.

### 2.4. Immunity

MIF acts on different immune cells and is implicated in the regulation of many immune pathways in cancer, particularly in melanoma (Figure 1).

Uveal melanoma represents a specific case in which the tumor cells act on the immune cells to survive. This melanoma subtype arises in the eye, which has a privileged immune environment, as innate and adaptive immune mechanisms are suppressed in the ocular environment. One of the mechanisms creating this immunosuppressive environment is the release of MIF leading to the inhibition of NK cell-mediated lytic activity. Hence, uveal melanoma cells are protected from immune destruction [34]. Repp et al. [34] showed that uveal melanoma cells release MIF while migrating to protect themselves against NK cell lysis. Indeed, 95% of uveal melanoma-related deaths are due to liver metastases, as the hepatic environment presents very high levels of NK cells [34]. They studied the supernatant of primary uveal melanoma tumors and metastases and showed that uveal melanoma metastases produced two-fold more MIF than primary uveal melanoma.

These observations indicate that certain melanoma cell types can secrete MIF to protect themselves against the immune microenvironment [35]. All these observations tend to prove that MIF can modulate the immune system.

Uveal melanoma is not the only type of melanoma to produce MIF. Indeed it has been indicated that many other melanoma cell types also produce MIF. In this way, they act on the activity of many immune cells. For example, Schoenfeld et al. [35] showed an attenuation of macrophage Tie-2 expression when they treated melanoma cells with anti-MIF antibodies. They also showed that patients with late stage melanoma in an immunotherapy trial, consisting of autologous GM-CSF secreting tumor cell vaccines and CTLA-4 blockade, developed auto-antibodies against MIF while they responded to their combined therapies [36].

MIF produced by the surrounding stroma also acts on circulating myeloid-derived suppressor cells (MDSCs) which are responsible for the monocytic immunosuppressive activity that appears in late stage melanoma patients. Yaddanapudi et al. [36] isolated circulating MDSCs from late stage melanoma patients and showed that these cells depend on MIF to suppress antigen-independent T-cell activation and to generate a maximal amount of ROS. This was also demonstrated in an in vitro model of co-culture of MDSCs and melanoma cells [37].

Another study, aiming to understand the antagonization of the anti-melanoma immune responses by MIF, revealed that, in melanoma bearing mice, MIF derived from macrophages participates in the macrophage alternative activation. Once again, they showed that MIF produced by the host matters more than MIF produced by melanoma cells. Indeed, they showed that peripheral macrophages and TAMs from melanoma models of MIF deficient mice had an elevated expression of pro-inflammatory cytokines and a reduced production of genes playing anti-inflammatory and immunosuppressive roles compared to melanoma models of wild-type mice. They also observed that TAMs and MDSCs from MIF-deficient mice had a reduced T lymphocyte immunosuppressive activity compared to TAMs and MDSCs isolated from wild-type mice. These phenomena led to a higher tumor immunosuppression by TAMs in MIF-deficient mice than in wild-type individuals [32].

Melanomas are highly immunogenic tumors. This is the reason why many immunotherapies have been studied to treat this cancer first. For example, the blockade of the CTLA-4 or PD-1 receptors with specific antibodies has shown encouraging results in clinical trials in patients with metastatic melanoma, despite a lack of efficiency for long term treatments [37,38]. The mechanism of resistance to such immunotherapies has been shown to be linked to an exacerbation of innate and adaptive immunosuppressive pathways by macrophages present in the tumor microenvironment and alternatively activated [39].

Yaddanapudi et al. [36] showed a promotion of alternative activation markers, and a reduction of classical activation markers, of peripheral macrophage polarization by MIF in mouse models of melanoma. They also showed that MIF inhibition reduces splenic MDSC immune suppression in melanoma mouse models, and is well-known that MDSCs act on immune-dependent and immune-independent mechanisms to promote tumor progression [32]. These observations, associated with other results showing that MIF neutralization in tumor bearing hosts induces cytotoxic T lymphocyte activities, suggest that MIF plays an important role in tumor-induced immune tolerance, both innate and adaptive, and immunosuppression in cancer, and more specifically in melanoma. The authors also showed that MIF inhibition led to a reduction of melanoma TAM and MDSC-mediated immune suppression [40].

Finally, Tanese et al. reported that the blockade of MIF-CD74 interaction reduces the expression of pro-tumorigenic molecules implied in immune and inflammatory mechanisms, such as interleukin-6 and interleukin-8 [41].

Thus, many studies have focused on the implication of MIF and the MIF-CD74 interaction on immunity. The results indicate that MIF acts on different immune pathways by regulating many immune molecules and cells, such as cytokines, MDSCs, cytotoxic T lymphocytes, and many types of macrophages (Tie-2, TAMs, etc.). By regulating all these factors, MIF is crucial in the development of immunotolerance and immunosuppressive mechanisms in the case of melanoma. All these observations tend to indicate that molecules targeting MIF should allow the restauration of immune mechanisms against melanoma cells.

Another important point regarding the link between MIF activity in melanoma and immunity concerns the production of interferon-γ (IFN-γ) by immune cells. Indeed, despite its crucial role in tumor immune surveillance, IFN-γ has recently been shown to enhance the expression of the CD74 receptor of MIF in melanoma. This regulation has been demonstrated in vitro by an increased transcription and cell surface expression of CD74 in melanoma cell lines following IFN-γ stimulation. In vivo, the inhibition of MIF-CD74 interaction in the presence of IFN-γ has been tested in xenograft mouse models, and a tumor growth inhibition has been observed. In patients, it has been observed that IFN-γ levels in plasma correlated with CD74 expression in melanoma tissues. These observations are independent of the BRAF or NRAS mutational status, demonstrating that this is a general phenomenon in melanomas [41].

### 2.5. Metastasis

Yaddanapudi et al. demonstrated that MIF deletion in melanoma-bearing mice protects them against lung metastatic colonization by improving the immunosuppressive response against melanoma cells and enhancing the peripheral macrophage pro-inflammatory responses. They also showed that MIF inhibition leads to a reprogramming of TAM polarization, which allows the attenuation of melanoma progression and pulmonary metastases [32]. These results indicate that the main effect exerted by MIF on metastasis development is linked to its modulation of immune mechanisms.

Clawson et al. [42] showed that melanocytes can be found in the blood of healthy individuals. These melanocytes express high levels of TYR and MITF, but not MLANA or MIF. When melanomas develop, the tumors can release factors which prevent these healthy melanocytes from entering the circulation, but instead melanoma cells expressing MLANA and MIF enter the blood circulation where they become circulating tumor cells (CTCs) which play a key role in metastasis development. This study also showed the interest of evaluating MLANA and MIF expression in circulating melanoma cells in order to identify early stage patients with more aggressive disease [42].

They also reported, in an additional study, that melanoma metastases can arise from the fusion of macrophages with tumor cells (MTFs). They studied these entities in the peripheral blood of patients with cutaneous melanoma and showed a wide expression of MIF inside these MTFs. Moreover, they showed that MTFs express a wide range of CXCR4 and CD44 which can interact with MIF and act on stem cell pathways. The production of CXCR4, CD44 and MIF by these entities plays a key role in the establishment of “niches” at distant sites [43].

These two last studies indicate that the production of MIF by circulating entities, either CTCs or MTFs, is crucial for their survival in the blood stream and their establishment in other organs, once again proving the role of MIF in metastasis development.

## 3. Future Therapies against MIF in Melanoma

There is no correlation between cell sensitivity to MIF depletion and the BRAF mutational status. Keeping in mind that many melanoma patients carry a BRAF mutation, and that they develop resistance to BRAF inhibitors, this observation is very interesting as MIF inhibitors could be used to treat many patients in relapse after a treatment with a mutant BRAF inhibitor [23]. Besides patients carrying a BRAF mutation, MIF inhibitors could technically be effective on many tumor types harboring different mutation profiles, providing therapeutic alternatives to patients whose tumors do not correspond to classical targeted therapies.

MIF silencing could be used as a new treatment targeting angiogenesis. Indeed, as shown earlier, MIF acts on TSP-1 and promotes angiogenesis. Experiments have shown that knocking down MIF expression in cells inoculated in mice led to similar-sized tumors establishing horizontally, but the vertical growth was strongly decreased, compared to mice inoculated with no-knock down melanoma cells. Furthermore, this decreased vertical growth was associated with a markedly decreased vascularization [28].

In addition, TAM polarization following MIF blockade is another source of proof that targeting MIF could help in restoring immune response, attenuating melanoma progression and pulmonary metastases in patients [32].

Many different strategies are already used in vitro and in preclinical studies to target MIF. These treatments include anti-MIF neutralizing antibodies, MIF-directed siRNA/shRNA, antisense oligonucleotides, and compounds acting on MIF secretion. These molecules have already given promising results, proving that targeting MIF could be effective in the treatment of many tumors, including melanoma [8,11,28,32]. But the main problem with these types of treatment strategies is that they are still far away from a possible clinical use.

Research is now focusing on more attractive approaches to decrease MIF activity by using small inhibitors, such as ISO-1 or ISO-66, which ligate the tautomerase active site of MIF, which plays a role in many of its biological functions. A study performed on colon cancer and melanoma showed that ISO-66 enhanced specific and non-specific immune responses in vitro, and its administration in vivo was nontoxic and resulted in decreased tumor burdens [44]. Regarding their lack of toxicity and their effectiveness on melanoma and colon cancer cells, these kinds of MIF inhibitors could be very promising for the future of cancer therapy, and especially melanoma treatment.

In addition, 4-iodo-6-phenylpyrimidine (4-IPP) is another small inhibitor acting on the tautomerase active site that could be used to treat patients with metastatic melanoma. This molecule is a small antagonist of MIF. A study performed on melanoma bearing mice showed that 4-IPP attenuates tumor polarized macrophage alternative activation, immunosuppression, neoangiogenesis, and melanoma tumor outgrowth [32].

An additional strategy is to indirectly target MIF by using Hsp90 inhibitors. Indeed, as MIF is a client of Hsp90 and is highly stabilized in many cancers, Hsp90 inhibitors could be a treatment of choice to reduce MIF levels. Studies have shown that pharmacological inhibition of Hsp90 activity destabilizes MIF in many different cancer cells. The major advantage of Hsp inhibitors is that constitutive Hsp90 upregulation is a tumor-specific characteristic, not present in normal cells, which should lead to reduced side effects [8,19,20,21,22,23,24,25,26,27,28,29,30,31,32,33,34,35,36,37,38,39,40,41,42,43,44,45,46]. Hsp90 inhibitors are not approved for a clinical use yet but there are over 25 active clinical trials (including 2 on melanoma) involving Hsp90 inhibitors [47,48]. It could be of importance to examine the role of MIF degradation in the response to Hsp90 inhibition. Another approach to target MIF could be the use of anti-MIF antibodies, which have already been developed (BaxG03 and BaxB0, for example). These antibodies are currently used in pre-clinical studies on many cancer types and have shown promising results on xenograft models of prostate cancer [45]. This suggests that a benefit could also be found in other cancer types.

Finally, more recently, a new promising approach to targeting MIF activities has been developed. This innovation uses a peptide-based therapy that allows the recovery of immune responses in metastatic melanoma. Indeed, a new 2018 study used the Id-CDR-based peptide, C36L1, which binds to the MIF receptor, CD74, to reactivate macrophages and dendritic cells in both in vitro and in vivo metastatic melanoma models [49].

All these data indicate that MIF targeting could be a therapy of choice in the future of melanoma treatment. Different research teams are trying to block MIF actions using different strategies such as small inhibitors, antibodies or peptides. For now, the most promising approach seems to rely on small inhibitors, such as ISO-66, which seems to be effective with minimal toxicity.

## 4. Conclusions

In the past few years, MIF has been largely studied in oncology for its major role in many tumorigenic pathways, such as angiogenesis, metastasis, and, most of all, immunity. Its role has been showed in many different cancer types. In this context, our review highlights that MIF could be a target of choice in metastatic melanoma treatment, where there is a significant lack of therapies after the development of resistance to targeted and immunotherapies.

Indeed, the results reported in this review indicate that MIF is produced by melanoma cells and has a prognostic value in the case of metastatic melanoma. MIF plays a key role in the development of metastatic disease by regulating angiogenesis and immunity, as well as by activating, through its binding to the CD74 receptor, the MAPK and the PI3K/AKT pathways, which are the most important signaling pathways in the development of melanoma. Conventional targeted therapies act on these two pathways and through its role on their upregulation, MIF could play a key role in the development of resistances to these gold standard therapies. Regarding this information, targeting MIF could help to reverse these resistance mechanisms.

Many studies are ongoing to target MIF and its receptors by various original strategies focusing on the use of antibodies, small inhibitors or even peptides. The current results seem promising both in vitro and in vivo, but they need to be validated in patients in combination with, or after resistance to, the gold standard treatments.
